# Dementia caregivers on Remo Health's Community Forum: A descriptive analysis

**DOI:** 10.1002/alz70858_106117

**Published:** 2025-12-24

**Authors:** Allison Pack, Christina Daniel, Christopher Vercammen‐Grandjean, William Poe, Matthew LeKrey, Jason Decastro, Meredith A Bock

**Affiliations:** ^1^ Remo Health, Cheyenne, WY, USA; ^2^ University of California, San Francisco, San Francisco, CA, USA

## Abstract

**Background:**

Alzheimer's disease and related dementias (**ADRD**) lead to progressive cognitive and physical decline. ADRD affects over 6 million adults over 65 in the US, with prevalence expected to double by 2050. Management of ADRD requires support from over 11 million unpaid caregivers, many of whom report increased stress, depression, and anxiety.

To address the needs of people with dementia (**PWD**) and their caregivers, Remo Health provides three services on its technological platform: Learn, a digital library of evidence‐based, expert‐reviewed content spanning the dementia journey; Community, a free, online caregiver community that offers 24/7 access to secure, bidirectional communication with dementia experts and others with similar lived experiences; and Clinical Care, interdisciplinary, comprehensive memory care provided via telehealth. This study describes data from caregivers on Remo Community.

**Method:**

To enter the Remo Community forum, caregivers must complete a brief electronic survey. We conducted simple descriptive statistics of recently added questions to better understand demographics, diagnosis and functional status of the PWD, and caregivers’ areas of greatest need.

**Result:**

During the study time period (12/9/24 to 1/24/25), 1,013 caregivers across 39 states newly signed up for Community and total members on Community reached 13,098. Most (69%) completed the new onboarding survey and reported mean age as 75 years (SD 12.5). “Alzheimer's” was the most commonly reported diagnosis (33%), followed by “no diagnosis” (26%). Perceived functional status ranged from “independent” (18%) to “dependent” (26%). Among those with “no diagnosis,” 21% reported the PWD is “dependent” for activities of daily living. Caregivers expressed their top needs as information on “caregiver coping and support” (42%), “brain health and wellbeing” (13%), and “dementia basics” (11%).

**Conclusion:**

In contrast to other studies, we found that older caregivers of PWD across the US can complete electronic surveys and seek information in online forums, particularly around coping with their responsibilities and understanding basic information about cognitive disorders. Results revealed dementia diagnosis must be prioritized, as caregivers reported many undiagnosed individuals they care for are functionally dependent. Future studies will incorporate clinical data and longitudinally examine the benefits of Remo Community for both caregivers and PWD.

